# EEG driving fatigue detection based on log-Mel spectrogram and convolutional recurrent neural networks

**DOI:** 10.3389/fnins.2023.1136609

**Published:** 2023-03-09

**Authors:** Dongrui Gao, Xue Tang, Manqing Wan, Guo Huang, Yongqing Zhang

**Affiliations:** ^1^School of Computer Science, Chengdu University of Information Technology, Chengdu, China; ^2^School of Electronic Information and Artificial Intelligence, Leshan Normal University, Leshan, China

**Keywords:** driving fatigue detection, EEG, convolutional neural network, recurrent neural network, log-Mel spectrogram

## Abstract

Driver fatigue detection is one of the essential tools to reduce accidents and improve traffic safety. Its main challenge lies in the problem of how to identify the driver's fatigue state accurately. Existing detection methods include yawning and blinking based on facial expressions and physiological signals. Still, lighting and the environment affect the detection results based on facial expressions. In contrast, the electroencephalographic (EEG) signal is a physiological signal that directly responds to the human mental state, thus reducing the impact on the detection results. This paper proposes a log-Mel spectrogram and Convolution Recurrent Neural Network (CRNN) model based on EEG to implement driver fatigue detection. This structure allows the advantages of the different networks to be exploited to overcome the disadvantages of using them individually. The process is as follows: first, the original EEG signal is subjected to a one-dimensional convolution method to achieve a Short Time Fourier Transform (STFT) and passed through a Mel filter bank to obtain a logarithmic Mel spectrogram, and then the resulting logarithmic Mel spectrogram is fed into a fatigue detection model to complete the fatigue detection task for the EEG signals. The fatigue detection model consists of a 6-layer convolutional neural network (CNN), bi-directional recurrent neural networks (Bi-RNNs), and a classifier. In the modeling phase, spectrogram features are transported to the 6-layer CNN to automatically learn high-level features, thereby extracting temporal features in the bi-directional RNN to obtain spectrogram-temporal information. Finally, the alert or fatigue state is obtained by a classifier consisting of a fully connected layer, a ReLU activation function, and a softmax function. Experiments were conducted on publicly available datasets in this study. The results show that the method can accurately distinguish between alert and fatigue states with high stability. In addition, the performance of four existing methods was compared with the results of the proposed method, all of which showed that the proposed method could achieve the best results so far.

## 1. Introduction

Mental fatigue is a psychobiological state caused by prolonged and demanding cognitive activity (Van Cutsem et al., 2017). This mental fatigue reduces a driver's ability to concentrate and make decisions, making it impossible to drive effectively. According to data released by China's National Bureau of Statistics, more than 60,000 people will die in traffic accidents nationwide in 2020 alone (Bureau, 2021). Traffic accidents cause great harm and loss to individuals, the country, and society. Therefore, fatigue detection has become an effective means of reducing accidents and improving transport safety.

To date, several widely used indicators have been proposed for detecting driving fatigue, such as monitoring fatigue-related facial expressions, blinking, yawning (Wang Z. et al., 2020), muscle (Zhang et al., 2021a), and measuring fatigue-related physiological variables such as electrooculography (Zheng and Lu, 2017), heart rate variability (Du et al., 2020a), and electroencephalography (Zhang et al., 2020). Of the many fatigue detection indicators, EEG signals are good mental indicators (Zhang Y. et al., 2022). Because the EEG signal is closely related to brain activity (Zhang X. et al., 2022). Therefore, this paper proposes a driving fatigue detection task based on EEG signals. In fatigue detection tasks, the mental state is usually classified into two categories of alertness and fatigue, or three categories of alertness, fatigue, and drowsiness. Several excellent research results have been presented to achieve this goal (Tuncer et al., 2021). However, current research on EEG driving fatigue detection is still at the point where only complex preprocessed EEG signals can be used for fatigue detection with good recognition results. Due to the instability of the EEG signal, it is challenging to obtain good results if these models are applied to different acquisition devices and scenarios. The task of inputting raw signals for fatigue detection is more challenging than preprocessed EEG models. The models should be better trained than the raw signals with the noise and artifacts removed. Unlike existing methods, the proposed approach in this paper focuses on extracting features from the raw signal for the driving fatigue detection task.

Traditional methods to implement EEG driving fatigue detection are mostly shallow models. A shallow model provides reasonable predictive power with minimal complexity. It consists of a few layers and requires limited training data. However, it requires predefined features with discriminative power. Artificial neural networks (ANNs) with a hidden layer and support vector machines (SVM) are well-known shallow models. Of these, ANNs have been widely used in EEG fatigue detection systems (Chai et al., 2014). In the literature (Vuckovic et al., 2002), to predict fatigue status from EEG signal, time series of inter- and intra-hemispheric cross-spectrogram densities of EEG signal are fed as input to an ANN, which then classifies driver status as either fatigue or alertness. In an alternative approach (King et al., 2006), the time domain EEG data is converted into frequency bands, delta, theta, alpha, and beta bands, and the frequency domain data is then fed to the ANN for fatigue detection. The literature (He et al., 2016) integrated multiple indicators of fatigue to build an ANN-based driver fatigue assessment model, where the EEG indicators were labeled as awake or fatigued. The average power spectrogram ratio A(theta + alpha)/beta for the theta, alpha, and beta bands was derived by fast Fourier transform and fed into the ANN for classifying the driver status as awake or fatigued. Alternatively, a Support Vector Machine (SVM) is specifically designed for the two-class problem. SVM has been employed in many fatigue detection systems to classify driver states according to different fatigue levels. The literature (Mu et al., 2017) extracted the spectrogram entropy, approximate entropy, sample entropy, and fuzzy entropy of EEG signals and fused them as feature vectors to feed into the SVM to classify driver fatigue states, and fusing the four feature entropies obtained the best classification results. In the literature (Yeo et al., 2009), the researcher trained the SVM for binary classification of fatigue states, with significant beta wave activity representing the alert EEG signals and a drop in the alpha wave representing the fatigued EEG signals, and the method obtained better classification results. However, the training set for shallow models is usually small, and when the dataset is too large, the model needs help building better classifiers and often relies on hand-extracted unique features. Deep learning (DL) models are widely used in fatigue driving detection to address this problem.

Recent research has shown that deep learning methods have yielded better results (Ed-Doughmi et al., 2020; Zhang et al., 2021b) and are widely used in various fields. Deep learning models incorporate a learned representation of the data rather than a task-specific approach. In contrast to shallow models, deep models can extract features from training data. Convolutional Neural Networks (CNN) (LeCun and Bengio, 1995) was the first deep model used for driver fatigue detection. Among them, the literature (Wu et al., 2021). The proposed 3D EEG signals-based CNN for driving fatigue classification has achieved remarkable success by projecting the 3D brain topology and its corresponding fatigue features into 2D space to form a brain fatigue topography fed into a coupled CNN-LSTM structure for fatigue classification. Wang H. et al. (2020) proposed an attention-based multiscale convolutional neural network combined with a graph convolutional network for driving fatigue classification, using a time-domain convolutional block to learn the features of each channel and a graph convolutional block network to learn the spatial filters. Finally, the features were passed to a classifier consisting of a softmax layer and a fully connected layer for fatigue classification of the EEG signal. Zeng et al. (2018) proposed two convolutional neural network models, EEG-Conv and EEG-Conv-R. The first model is a conventional CNN. The second combines a CNN with a deep-learning residual network for driver fatigue classification. The results show that both network models outperform the classifier based on the support vector machine. The convolutional neural network combined with the residual network has better generalization capability. Du et al. (2020b) developed a TK-type Convolution Recurrent Fuzzy Network (TCRFN). This method uses convolutional neural networks to deal with noise and improve fatigue classification, in which the authors projected the three-dimensional coordinates of electrodes of EEG and fatigue-related frequency bands theta, alpha, pre-beta, post-beta, gamma onto a two-dimensional plane so that the EEG data is converted into a series of images as feature vectors, the literature concludes that convolutional neural networks are effective in reducing the effect of noise on the model TCRFN. However, due to the massive amount of EEG data, the current methods for extracting EEG signal features may overlook various important information. To solve the above problems, this paper proposes a model that combines spectrogram feature and temporal feature information, which can effectively utilize time-frequency information to improve the accuracy and precision of fatigue detection. Due to the many hyperparameters and complex structures involved, many DL structures often suffer from time-consuming training processes. We adjust the hyperparameters and model structure to achieve better results in terms of both training time and classification results.

The main contributions of this paper are as follows. (1) In the feature extraction stage, the traditional Fourier transform calculation method is abandoned, and a one-dimensional (1D) convolutional neural network is used to implement the short-time Fourier transform, obtaining more discriminative features. (2) Application of the speech signal technique log-Mel spectrogram to EEG signals to extract the time-frequency characteristics of EEG signals. (3) Combining convolutional neural networks and bi-directional recurrent neural networks to learn the high-level features of the signal, the implementation results show that adding a bi-directional recurrent neural network to the model yields the current optimal results.

The paper is structured as follows. Section 2 describes the related work in this paper. In Section 3, introduce the driving fatigue dataset. Section 4 describes the proposed method and feature extraction. In Section 5, experimental results are given, and the four existing methods are compared with the method proposed in this paper. Finally, Section 6 concludes with a summary and an outlook of the new model in practical driving applications.

## 2. Related work

This paper's feature extraction of the proposed method mainly involves the Short Time Fourier Transform and the log-Mel spectrogram. Therefore, this section focuses on the fatigue detection research work related to the short-time Fourier transform and log-Mel spectrogram and the proposed method and contributions.

Since EEG signals contain rich brain function information, many research methods can analyze EEG signals with spectrum technology. The literature (Kıymık et al., 2005) applied short-time Fourier transform (STFT) and wavelet transform (WT) to the EEG signal of normal children and children with epileptic seizures. The results showed that STFT has a short processing time and is more suitable for the real-time processing of EEG signals. In the methods (Sparto et al., 2000), the authors used STFT and wavelet transform to process surface EMG signals from the medial, lateral, and latissimus dorsi sites of the erector spinae. The results showed that both methods could detect and quantify fatigue. In another paper (Hajinoroozi et al., 2016), the authors used the fast Fourier transform (FFT) with a Hamming window to obtain frequency features and associated eigenvectors. Numerous advances in brain-computer interface (BCI) technology have demonstrated the feasibility of classification methods based on the short-time Fourier transform. Therefore, in this paper, we will use the short-time Fourier transform to extract the spectrogram features of EEG signals. Still, unlike traditional methods, we use a novel one-dimensional convolutional neural network to implement the Fourier transform work.

Another part of the extraction of features is the extraction of the signal's log-Mel spectrogram. Mel Spectrogram (Li et al., 2001) was first applied to the research of speech recognition. Due to the nonlinearity of the signal and the relationship between the time-frequency domain, the Mel filter bank or Bark filter bank method is usually used, and the extracted spectrogram can be used in speech recognition. The Mel spectrogram can represent the frequency energy independent of the input signal source. Other research areas use log Mel spectrograms to extract features and for deep learning classification tasks. In the literature (Dehzangi and Taherisadr, 2018), the authors proposed a system for detecting distracted drivers based on galvanic skin response (GSR) detection, which converts one-dimensional EEG signals into a two-dimensional spectrogram feature map by extracting the Mel spectrogram of the original GSR as a feature, and obtains good classification results. The literature (Kumar et al., 2021) removes the Mel spectrogram, STFT, and Croma of the emotional speech signal as input to the model. The method experiments with each of the three features. The experimental results show that the proposed model gets the best results using the Mel spectrogram features. In the method (Woo et al., 2022), sleep stage classification based on single-channel EEG signals was studied using the frequency-domain feature extraction method Mel Spectrum. Experiments show that using the Mel spectrogram, the number of input samples will be significantly reduced, the neural network training will be accelerated, and more discriminative features will be obtained. In the literature (Meng et al., 2019), an algorithm based on a 3D log Mel spectrogram is proposed for speech emotion recognition. Experimental results show a 4.58% improvement in recognition accuracy and processing time, showing that log Mel spectrogram maps are practical features for classification tasks. Therefore, we applied the log-Mel spectrogram features to the EEG-based fatigue classification task. The addition of the log-Mel spectrogram improved the accuracy of fatigue detection. The relationship between the learned features and fatigue information could be established through the fatigue classification model once the feature extraction was completed.

## 3. The proposed model

### 3.1. Feature extraction

Feature extraction takes the most relevant information from the original data and assigns that information to a lower dimensional space. When the input data is too large and not informative, the data is considered redundant. The input data is then transformed into a simplified representation of the features, also known as a feature vector. This conversion process is called feature extraction. Classification is performed based on the selected features. The classifier's performance depends on the signal's quality and the soundness of the feature selection. As the EEG signal has significant features in the time-frequency domain, to obtain the feature vector set, this paper implements the STFT by using one-dimensional convolution to get the signal's spectrogram, then uses the Mel filter bank to obtain the Mel spectrogram. A spectrogram is a visual representation of a signal's frequency spectrogram as a time function. The STFT can capture local features accurately, similar to a convolutional filter. STFT is the essential operation for calculating the Mel Spectrogram. To convert the STFT spectrogram to a Mel Spectrogram, the spectrogram is multiplied by the Mel filter bank kernel. We then explain using a 1D convolutional neural network to compute the STFT and extract the Mel spectrogram using a Mel filter bank.

#### 3.1.1. STFT

We implement STFT using a 1D convolutional network, where the convolutional kernel is initialized as the product of a DFT matrix and a window function in a 1D convolutional operation. The DFT matrix is an n*n matrix that the following equation can express.

(1) $$\begin{array}{l} \{\begin{array}{c} {\left(F\right)}_{nm}=\omega^{-nm}\\ \omega =e^{2\pi i\frac{1}{N}} \end{array} \end{array}$$

Where *nm* is the matrix of the n columns. In this paper, we apply the Hanning window function, which is calculated in the following form:

(2) $$\begin{array}{l} \omega \left(n\right)=\frac{1}{2}\left[1-\cos\left(\frac{2\pi \left(n-1\right)}{N}\right)\right],0\leq n\leq N-1 \end{array}$$

Where *n* denotes the total length of the window function, and *N* represents the effective length of the window function. The size of the convolution kernel is equal to the transform size of the STFT, and the step size is the hop length. The EEG signal is an actual signal, so the real and imaginary parts obtained can be seen as the result of the separate action of the real and imaginary parts of the convolution kernel, expressed as:

(3) $$\begin{array}{l} \{\begin{array}{l} Y^{R}=Conv1d\left(x,F_{DFT}^{R}⊙w,S\right)\\ Y^{I}=Conv1d\left(x,F_{DFT}^{I}⊙w,S\right) \end{array} \end{array}$$

where *x* is the EEG input signal, *w* is the window function, and *S* is the step size. A convolution operation is synthesized to obtain the signal after STFT:

(4) $$\begin{array}{l} STFT\left(x\right)=\left[Y^{R};Y^{I}\right]=Conv1d\left(x,{\left[F_{DFT}^{R};F_{DFT}^{I}\right]}^{T}⊙w,S\right) \end{array}$$

At this point, the returned STFT is in the form of a full FFT. For general feature extraction, only the first *N*/2 + 1 part of the DFT matrix is taken when initializing the convolution kernel. To obtain the spectrogram, the above equation also needs to be squared.

(5) $$\begin{array}{l} Spectrogram\left(x\right)={\left[Y^{R}\right]}^{2}+{\left[Y^{I}\right]}^{2} \end{array}$$

There are two main advantages of using a short-time Fourier transform based on a one-dimensional convolutional neural network. First, it supports batch processing. Using a neural network-based framework, we can enable tensor operations to convert a tensor of EEG signal segments into a tensor of spectrograms. Secondly, it is trainable. We will discuss how the prediction accuracy of the model can be improved by adjusting the number of convolution kernels of the one-dimensional convolution.

#### 3.1.2. Log-Mel spectrogram

In order to extract the unique perceptual features from the EEG signal, this work further extracts the signal's Mel spectrogram based on the spectrogram for use in a deep learning system for fatigue detection. The traditional frequency-to-Mel scale conversion is in D. O'shaughnessy's book (Douglas, 2000), as follows.

(6) $$\begin{array}{l} Mel\left(f\right)=2595\log_{10}\left(1+\frac{f}{700}\right) \end{array}$$

Once the conversion from frequency to Mel scale has been achieved, we can create the Mel filter bank. The equation to implement the mth filter of the filter bank is expressed as:

(7) $$\begin{array}{l} \{\begin{array}{c} 0,k<f_{m-1}andk>f_{m+1}\\ \frac{k-f_{m-1}}{f_{m}-f_{m-1}},f_{m-1}\leq k\leq f_{m}\\ \frac{f_{m+1}-k}{f_{m+1}-f_{m}},f_{m}\leq k\leq f_{m+1} \end{array} \end{array}$$

Where *f* is the Mel-scale frequency, and *m* is the total number of filters in the band. These filter banks were multiplied with the spectrograms of the results obtained by STFT above to obtain the Mel-scale spectrograms, as follows.

(8) $$\begin{array}{l} MS\left(x\right)=Spectrogram\left(x\right)⊙B_{m}\left(k\right) \end{array}$$

We also need to convert the power spectrogram to dB units by performing a logarithmic operation to obtain a log-Mel spectrogram. The purpose of taking the logarithm is to have the low-amplitude components pulled higher relative to the high-amplitude components to observe periodic signals masked by low-amplitude noise, as shown in the logarithm below:

(9) $$\begin{array}{l} LogMelSpec=10*\log_{10}\left(MS\left(x\right)\right)-10*\log_{10}\left(ref\right) \end{array}$$

where *ref* is the reference value by which the amplitude *MS*(*x*) is scaled relative to *ref*. The extracted log-Mel spectrogram features are fed into the model for the next step of training. The details of the model proposed in this paper are presented below.

### 3.2. Model description

CNN's have been widely used in EEG-based classification, such as driving fatigue detection (Wang H. et al., 2020; Wu et al., 2021). A convolutional neural network consists of several blocks, with one convolutional block composed of several layers of convolutional layers. High-level local features are obtained from the input feature vector. The log-Mel spectrogram features extracted above are used as input to the CNN.

#### 3.2.1. CNNs

The six-layer CNN proposed in this paper consists of three convolutional blocks, derived from a VGG-like CNN (Simonyan and Zisserman, 2015) perception. Each convolutional block consists of two convolutional layers with a kernel size of 3 × 3, a step size of 1 × 1, and a padding of 1 × 1. Batch normalization is applied between each convolutional layer (Ioffe and Szegedy, 2015). The mathematical definition of continuous convolution is given in Equation (12) and, in the discrete case, in Equation (13).

(10) $$\begin{array}{l} f*g\left(n\right)=\int_{-\infty }^{+\infty }f\left(\tau \right)g\left(n-\tau \right)d\tau \end{array}$$

(11) $$\begin{array}{l} f*g\left(n\right)=\overset{+\infty }{\underset{\tau =-\infty }{\sum }}f\left(\tau \right)g\left(n-\tau \right) \end{array}$$

where *f* and *g* do convolution operations. Suppose the batch normalization layer is not used. In that case, it will easily lead to the model's training to reach the activation function's gradient saturation zone. This is because when the network reaches a certain depth and has a certain complexity, the accumulated changes in the underlying network will affect the upper network, and the normalization operation can make the input data of the activation function fall in the gradient non-saturation zone and alleviate the problem of gradient disappearance. Finally, a 2 ∗ 2 averaging pooling layer is used for downsampling, and the literature (Kong et al., 2019) demonstrates that 2 ∗ 2 averaging pooling is a better choice than 2 ∗ 2 max pooling. A dropout is applied between each convolutional block (Hinton et al., 2016) regularization technique, which is mainly used to avoid complex mutual adaptation on the training data to combat overfitting in the network, with probability p set to 0.2, i.e., we drop neurons in the convolutional layer with probability 0.2.

**Figure 1:**
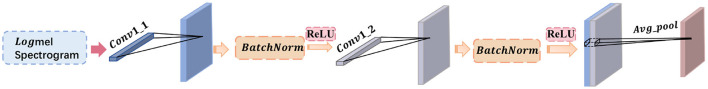
Schematic diagram of the convolutional block model.

The signal of 17 channels will be applied with a sliding window size of 1,600 to segment the data. After a small amount of trial and error, the patch size in training is finally set to 200. Each segment will get a sequence of eight small frames. Each frame will be input to the CNNs after extracting the above log-Mel spectrogram features. The next spliced output feature vector will be the extracted high-level features *X*. *X* will be used as the input to the recurrent neural network. The convolutional block model is shown in Figure 1.

#### 3.2.2. LogMel-CRNN

In this work of extracting temporal features, we choose a recurrent neural network that captures temporal relationships, which is an end-to-end model that is good at processing time series, such as the EEG signal or speech signal we use, that is, a sample where the preceding and following inputs are correlated. RNNs can be used to extract high-level features in the temporal domain. Schuster and Paliwal (1997) proposed a bi-directional RNN, which uses information from both ends of the sequence to estimate the output since the current value of the sequence depends not only on the information of the previous sequence but also on the sequence at the future moment so that the RNN structure can capture more of the long-term dependence of the sequence and obtain more information about the sequence, which is beneficial to the classification results. In this study, we constructed a two-layer bi-directional RNN with 128 hidden cell counts to model features in the time domain, with the aim of exploring the intrinsic relationships between consecutive time sequences. The values of forward and backward propagation determine the output values of a bi-directional RNN. A deep bi-directional RNN is shown below, denoted by *i* denoting the ith layer.

(12) $$\begin{array}{l} \{\begin{array}{c} s_{t}^{\left(1\right)}=f\left(U^{\left(1\right)}x_{t}+W^{1}s_{t-1}^{\left(1\right)}+b^{\left(1\right)}\right)\\ s_{t}^{{\;}^{\prime }\left(1\right)}=f\left(U^{{\;}^{\prime }\left(1\right)}x_{t}+W^{{\;}^{\prime }\left(1\right)}s_{t-1}^{{\;}^{\prime }\left(1\right)}+b^{{\;}^{\prime }\left(1\right)}\right)\\ \cdot \cdot \cdot \\ s_{t}^{\left(i\right)}=f\left(U^{\left(i\right)}s_{t}^{\left(i-1\right)}+W^{i}s_{t-1}^{\left(i\right)}+b^{\left(i\right)}\right)\\ s_{t}^{{\;}^{\prime }\left(i\right)}=f\left(U^{{\;}^{\prime }\left(i\right)}s_{t}^{{\;}^{\prime }\left(i-1\right)}+W^{{\;}^{\prime }\left(i\right)}s_{t+1}^{{\;}^{\prime }\left(i\right)}+b^{{\;}^{\prime }\left(i\right)}\right)\\ o_{t}=g\left(V^{\left(i\right)}s_{t}^{\left(i\right)}+V^{{\;}^{\prime }\left(i\right)}s_{t}^{{\;}^{\prime }\left(i\right)}+c^{\left(i\right)}\right) \end{array} \end{array}$$

Of these, the *U*, *V*, *W* and *U*′, *V*′, *W*′ are both weight matrices, and *b*, *b*′, *c* is the weight vector. *s*_*t*_ indicates that a forward calculation is being performed, and $s_{t}^{{\;}^{\prime }}$ prime is the inverse calculation, and *o*_*t*_ is the value of the output layer. For deep bi-directional RNNs, the more layers, the better the learning ability, but this requires more training data. In summary, the features extracted by the convolutional neural network are fed into the bi-directional RNN to obtain time-frequency domain features, which then enter the classifier, which consists of two fully connected layers and softmax functions, with the final predictions defined as follows.

(13) $$\begin{array}{l} Prob=Softmax\left(W_{s}\times F+b_{s}\right),Prob\in R^{z} \end{array}$$

Where $W_{s}\in R^{z\times l}$ is the weight matrix, and $b_{s}\in R^{k}$ is the bias vector, *l* denotes the size of the fully connected layer, *z* is the number of classifications in the model, and *k* represents the dimension of the feature *F*.

**Figure 2:**
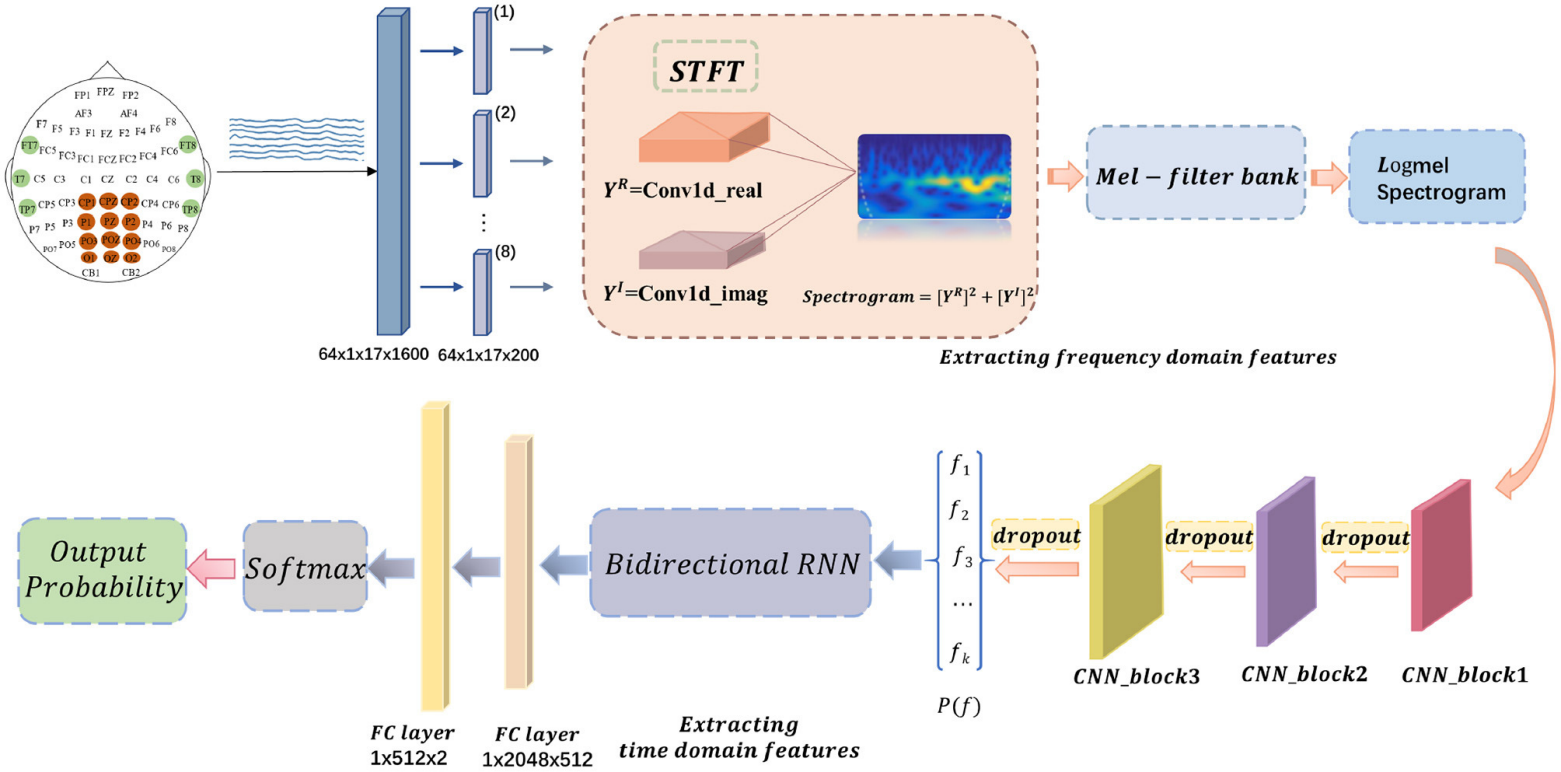
Schematic diagram of the whole model of the proposed LogMel-CRNN model.

Overall, in this paper, the extracted frequency domain features are fed into a six-layer CNN to extract high-level features, which are then provided into a two-layer bi-directional RNN. The resulting temporal features are finally fed into a classifier consisting of a fully connected layer, a ReLU activation function, and a softmax function to produce the final classification prediction results. The schematic diagram of the proposed model, LogMel-CRNN, is shown in Figure 2.

## 4. Experiments and analysis of experimental results

### 4.1. Datasets

Experimental paradigm: This study used the SEED-VIG (Zheng and Lu, 2017) dataset from the Human Brain Computing and Machine Intelligence Commons at Shanghai Jiao Tong University. The experiments were based on EEG signals collected by a virtual reality driving simulation system, in which each participant was tested for ~2 h. Collect EEG signals: 21 EEG data sets were collected from 23 volunteer participants. Twelve channels of EEG signals were recorded from posterior sites (CP1, CPZ, CP2, P1, PZ, P2, PO3, POZ, PO4, O1, OZ, and O2), and six channels of electrical signals were recorded from temporal sites (FT7, FT8, T7, T8, TP7, and TP8). One of the electrodes, CPZ, was the reference electrode. Raw EEG signals were acquired from 18 channels at a sampling rate of 1,000 Hz. Labeling: The SEED-VIG dataset includes both EEG and EEG signals. Where the labels were obtained using SensoMotoric Instruments (SMI) eye-tracking glasses, the data were labeled as the percentage of eyelids closed over the pupil over some time, PERCLOS (Gao et al., 2015), which was calculated as follows:

(14) $$\begin{array}{l} PERCLOS=\frac{blink+CLOS}{interval} \end{array}$$

(15) $$\begin{array}{l} interval=blink+fixation+saccade+CLOS \end{array}$$

Among them, *blink* is the blink time, fixation is the gaze time, a saccade is the saccade time, and *CLOS* is the closed eye time. PERCLOS values were classified into three categories: wakefulness, fatigue, and somnolence, through thresholds of 0.35 and 0.7. The smaller the PERCLOS value, the higher the driver's alertness. In this paper, two groups of experiments are done. One is that the binary classification is divided into alertness and fatigue state through the threshold of 0.35; the second is that the multi-class category is divided into the state of alertness, fatigue, and sleepiness through the thresholds of 0.35 and 0.7.

### 4.2. Experimental settings

During the initial training of the experiments, the Xavier normal distribution was used to initialize the network weights. LogMel-CRNN used the Adam optimizer and a learning rate set to 1.0e-4. To perform a comprehensive evaluation of the model, the leave-out validation method was used to obtain the model with the highest accuracy. After disrupting the dataset, the ratio of the training set to the test set was 8:2. Considering the training time of the network, the batch size was set to 64 when training the network, and 50 epochs were required. The cross-entropy function was the model's loss function and was calculated as follows.

(16) $$\begin{array}{l} CE=\overset{K}{\underset{k=1}{\sum }}-P_{k}\log\left(p_{k}\right) \end{array}$$

Where *P* is the true distribution, which is a one-hot vector of length *K* a one-hot vector of length *P*_*k*_ ∈ (0, 1), *p* is the predictive distribution.

We applied four standard metrics to measure the performance of our model from distinct perspectives: Accuracy indicates the precision of the prediction results; Precision suggests the probability of correctly predicting a positive sample among the samples predicted to be positive; Recall suggests the probability of being correctly predicted as the last positive sample among the positive samples of the original sample; F1- Score is the summed average of precision and recall.

### 4.3. Model ablation

**Figure 3:**
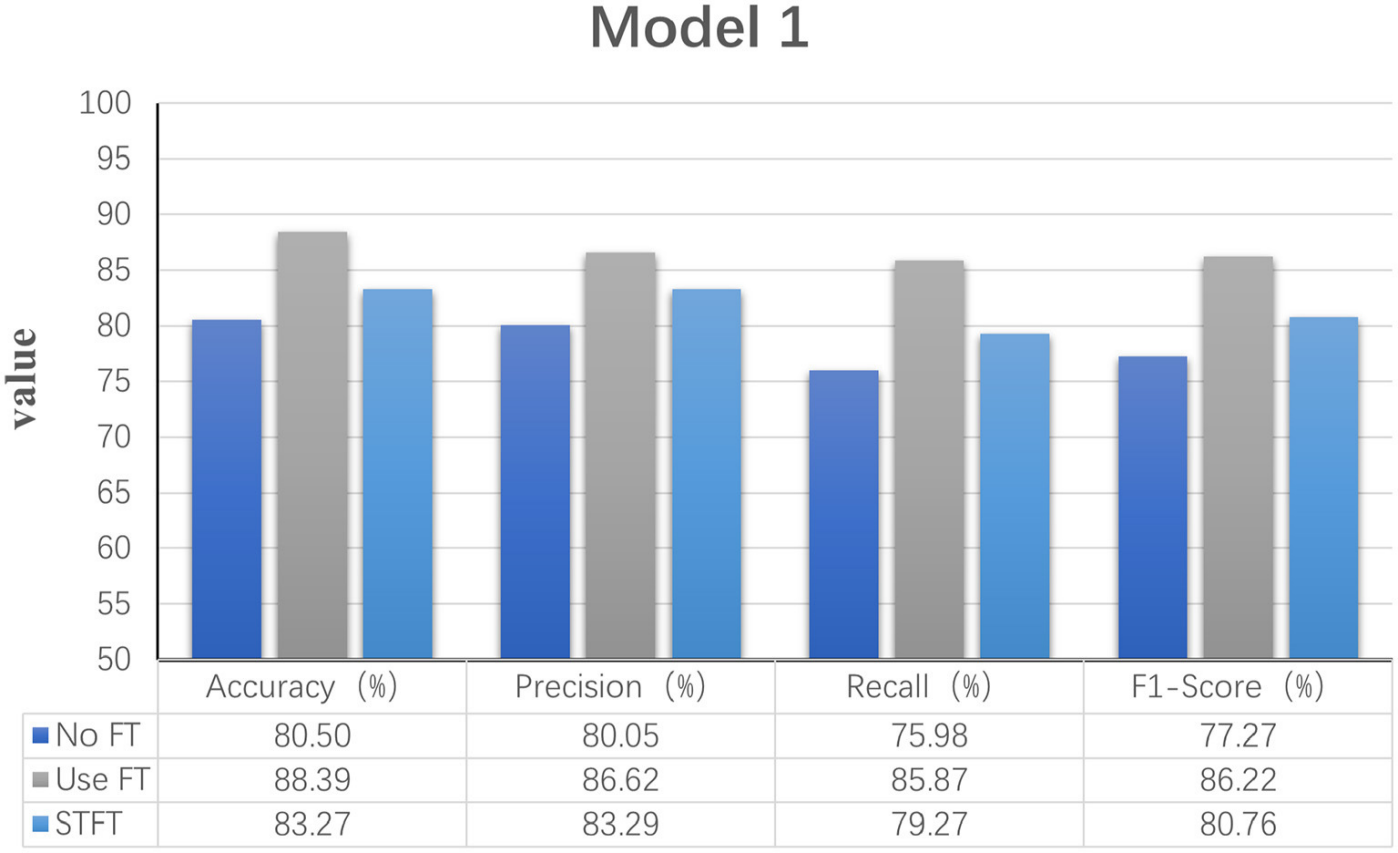
Statistical graphs of the evaluation results of the model with the addition of FT vs. without FT.

**Figure 4:**
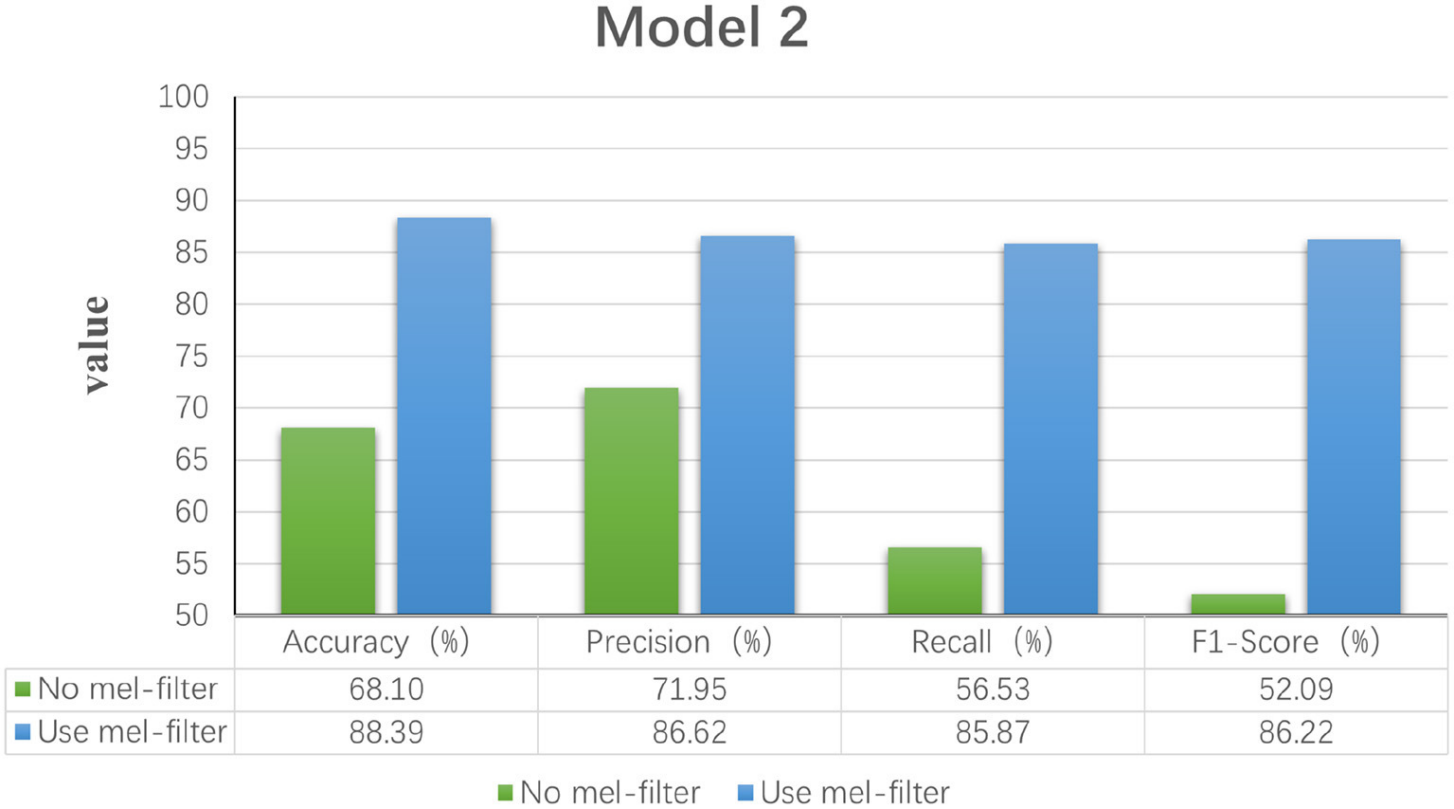
Statistical graphs of the evaluation results of the model with the addition of Mel filter vs. without.

**Figure 5:**
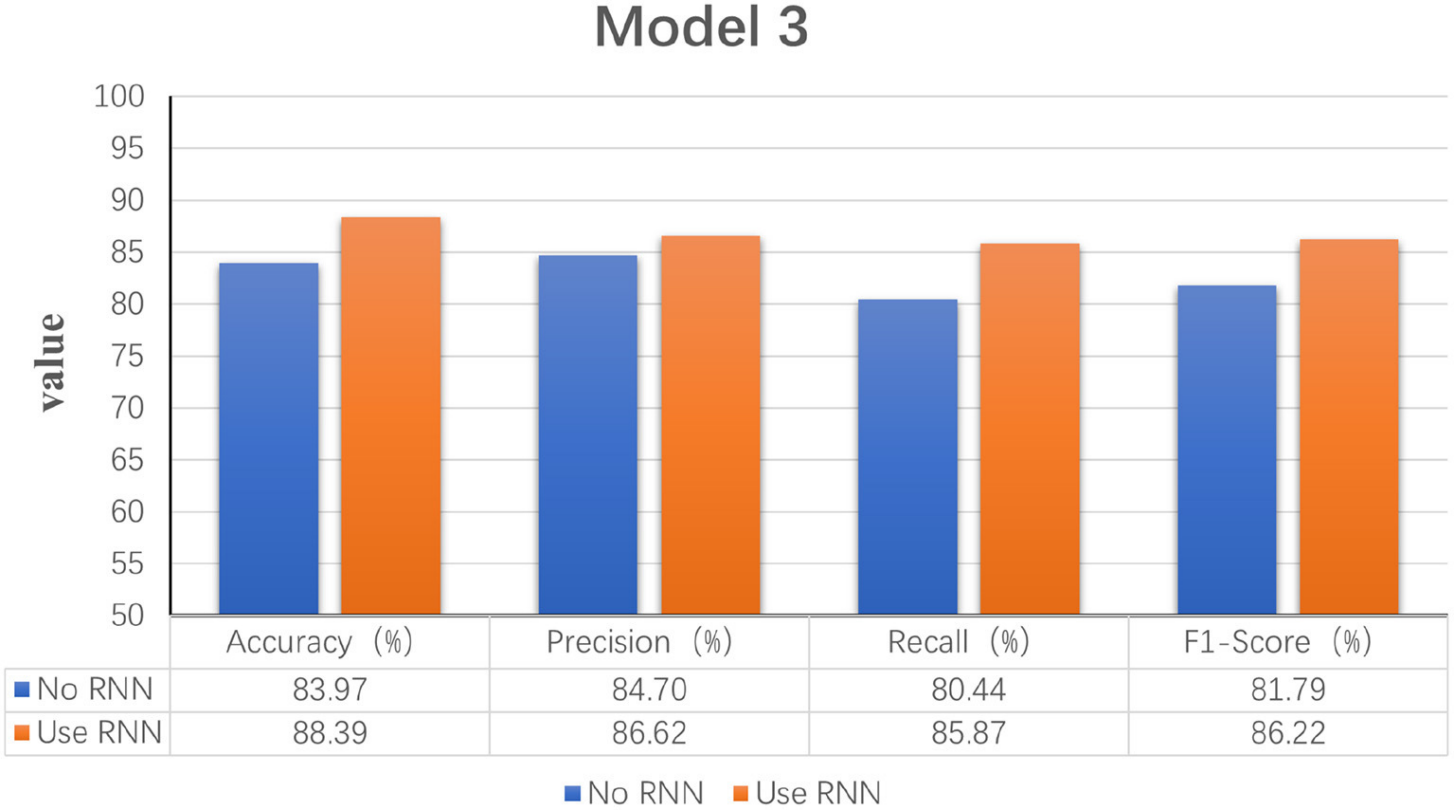
Statistical graphs of the evaluation results of the model with the addition of RNN vs. without RNN.

**Figure 6:**
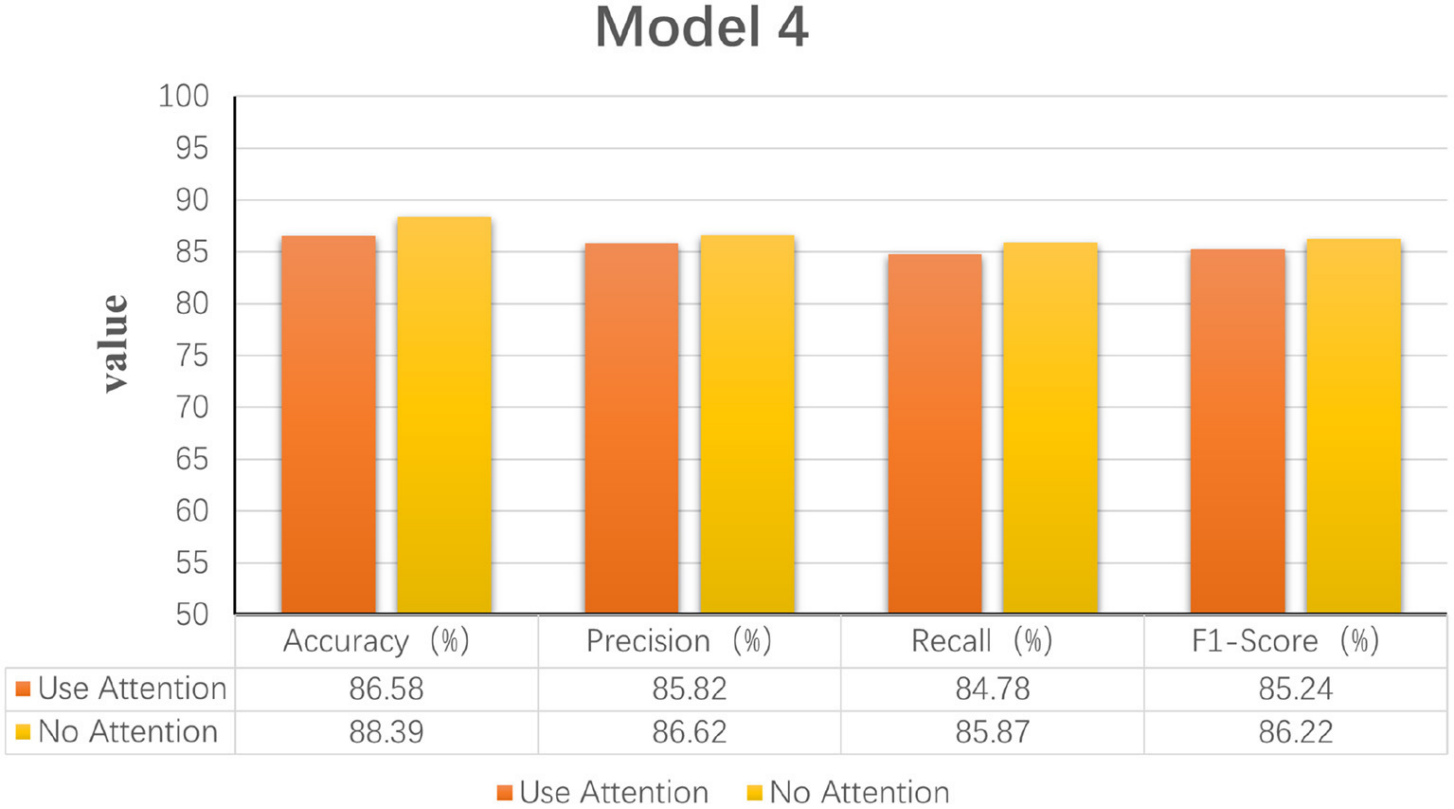
Statistical graphs of the evaluation results of the model with the addition of attention mechanism vs. without.

To study the utility of each component in our model, we decomposed our proposed LogMel-CRNN model with a set of variants, including the following:

1. Model 1: Fourier transforms and comparing Fourier transform types
  In order to explore the effect of frequency domain information on feature extraction, in the model LogMel-CRNN basis, the model with frequency domain information obtained by the Fourier transform is compared with the model without FT, and the results show that the performance indicators of the former results are all greater than those of the latter. And the comparison will be made with the extraction of frequency domain information using the conventional STFT, which performs less well than the STFT using one-dimensional convolution, which is consistent with the previous (Kumar et al., 2021) experimental results of extracting frequency domain information to obtain better classification results that are consistent. Figure 3 shows the results of the experimental comparison of the average evaluation metric with and without the Fourier transform and the experimental classification evaluation results using the conventional short-time Fourier transform. In Figures 3–**6**, the abscissa is the four classification indicators, the ordinate is the evaluation result value, and the results are averaged. The graph shows that the model using the Fourier transform implemented using one-dimensional convolution achieves the highest accuracy, with the remaining three indicators being higher than the other two experiments.
2. Model 2: A Mel filter bank
  The Mel filter bank converts frequencies to a Mel scale, which results in a Mel spectrogram. The Mel scale relates the perceived frequency of the original signal to the actual measured frequency. Humans are much better at recognizing small pitch changes at low frequencies than at high frequencies (Wu and Cao, 2005). So the Mel scale makes the features much closer to what humans perceive. In this model, we verified that extracting the log Mel spectrogram plays a positive role in the model. The model has better robustness when it is used compared to the model without the Mel filter bank. Figure 4 shows the results of the experimental classification evaluation of the model with and without the Mel filter bank. The classification results are much higher for the experiments with the model with the addition of the Mel filter set than for the experiments without it. In particular, the F1 score is even higher than 34.13%. This shows that the Mel filter bank is able to extract significant fatigue features.
3. Model 3: Bi-directional RNN
  This experiment aims to determine whether the time domain contains valuable information. In this study, a bi-directional RNN was used to extract temporal features. The RNN has two hidden layers, each with a hidden cell size of 128. We find a relationship between temporal order and fatigue classification as time changes. The experimental results show that using RNNs to extract features and then feeding them into the classifier achieves better classification results. Figure 5 shows a plot of the evaluation results of the experimental results of the model with and without the RNN. It can be seen that adding the RNN module resulted in a 4.42% increase in fatigue classification accuracy precisely because the RNN can provide learning training using temporal information to improve model performance.
4. Model 4: Increased attention mechanism
  In this study, we also experimented with the use of attention mechanisms after extracting temporal features. The attention mechanism in deep learning borrows from the four-dimensional approach to human attention and has been used in a variety of different types of deep learning tasks, such as natural language, image classification, and speech recognition, with significant results. However, the experimental results did not show satisfactory results, so the attention mechanism module was not used in the model. Figure 6 shows the experimental evaluation results of adding the attention mechanism to the model versus not employing it. As seen in the table, the classification results with the attention mechanism will be slightly worse than the model without it. Therefore, the final LogMel-CRNN model did not adopt the attention mechanism.

### 4.4. Influence of important parameters

In this section, we studied the influence of five parts: (1) The number of STFT-1D convolutional kernel size. (2) The size of the STFT-Hop length of our model. (3) The number of Mel filter banks. (4) The sampling rate of Mel filter banks. (5) The patch size of CNN model.

1. STFT-1D convolutional kernel size
  In the STFT calculation, this is achieved by one-dimensional convolution, in which the size of the convolution kernel is equal to the size of the FT (n_fft). The experiments are performed by adjusting the n_fft parameter to change the perceptual field during the convolution. Therefore, this parameter directly changes the output of the frequency spectrogram and is an essential parameter for this experiment. Table 1 shows the classification evaluation indicators after four parameter adjustments. The first column of the table shows the size of the set convolution kernel parameters. The first row shows the four average evaluation indicators. Each row indicates the average evaluation of the classification results at that convolution kernel size. Obviously, in the four adjustment parameters, when the size of the convolution kernel, that is, the size of the FT, is 50, the model achieves better results, with an accuracy rate of 88.39%. When the convolution kernel size is 100, the accuracy rate also reaches 87.34%. The table shows that the method of implementing the short-time Fourier transform using one-dimensional convolution makes the short-time Fourier transform trainable.
2. STFT-Hop length
  Hop length is the second important parameter in the STFT calculation. It is the distance that the window moves, also known as the frameshift. The window size for this experiment was set to 10 samples, and the window was changed to overlap or not by adjusting the window shift distance. When the Hop length is five samples, which is equal to one-half of the window size, there is an overlap of five frames. Table 2 shows the average evaluation indicators obtained after adjusting the hop length four times. The first column of the table shows the set hop length, the first row shows the four average evaluation indicators, and each of the remaining rows shows the results of the evaluation indicators for that hop length size. The table shows that the best classification accuracy was obtained when the Hop length size was 1, while the F1 score got the best result of 86.37% when the Hop length was 5.
3. Mel filter banks - number of filters
  The final step in calculating the filter banks is to apply the triangular filter on the Mel scale to the power spectrogram to extract the frequency bands, mapping n_fft into the mel bin. According to Equation (9), after the filter bank has been created, the first filter bank will start at the first point, peak at the second point, and then return to 0 at the third point. The second filter bank will begin with the second point, reach a maximum at the third point, then be 0 at the fourth point, and so on. To obtain a Mel spectrogram plot with 12 mel bins, we would need 12 Mel filter banks. Table 3 shows the average evaluation indicators for the three different numbers of Mel filter banks set up for this experiment. The first column of the table shows the number of Mel filters set up, and the first row shows the four different evaluation indicators. It is clear from the table that the four evaluation indicators for fatigue classification are much higher than the other two groups when the number of Mel filters is 12. This shows that the number of Mel filters is an essential parameter for the model's training.
4. Mel filter banks - sampling rate
  The sampling rate parameter of the Mel filter bank, i.e., the sampling rate of the input signal. The sampling rate of the original EEG signal was 1,000 Hz, and the best input sampling rate parameter was obtained by five experiments of adjusting the input signal's sampling rate. Table 4 shows the results of the average evaluation metric for the five experiments. The first column indicates the size of the set sampling rate, and the first row shows the four different evaluation indicators. As can be seen from the table, the four indicators for fatigue classification are higher than the other four experiments when the input signal has a sampling rate of 400 Hz.
5. CNN-Patch size
  Patch size: Previous research has shown that using a larger patch size in a CNN can result in better classification accuracy because the CNN can capture more contextual information to make decisions (Farabet et al., 2012; Li et al., 2014). This is because CNNs can capture more contextual information to make decisions. Therefore, testing a larger network and increasing the patch size also requires changes to the network, as some layers (e.g., fully connected layers) are, by definition, required to have a fixed-size input. So, a patch size change also implies a layered architecture change. Table 5 shows the results of the average evaluation metric for three experiments with the Patch size parameter adjusted. The first column of the table sets the size of the patch size and the first row shows the four average evaluation indicators for fatigue classification. From this table, the best precision value of 86.62% was obtained for a patch size of 200 in the convolutional neural network in this experiment. The performance of the model was also improved and the evaluation indicators of the model were optimized.
  After five parameter tuning experiments, the best parameter combinations for this study are summarized in Table 6, where the left-hand side of the table indicates the names of the different parameters, and the right-hand side shows the optimal values for each parameter. Table 7 provides the results of the average fatigue assessment metric for the two-classification hybrid experiment of LogMel-CRNN, with an accuracy of 88.39%, a precision of 86.62%, recall of 85.87%, and an f1 score of 86.22% for the two-classification. Table 8 shows the results of the average assessment indicators for fatigue for the three-classification mixed experiment of LogMel-CRNN. As seen in this table, accuracy is 81.30%, precision is 81.67%, recall is 81.97%, and f1 score is 81.80%. The assessment indicators for the three-classification experiment were much lower than those for the two-classification because the EEG signals for fatigue and drowsiness in the three-classification were very similar. The model needed help to distinguish the significant difference between the two.

**Table 1:** Average performance results based on differences in the size of the convolution kernel for 1D convolution in the Fourier transform.

**Kernel size**	**Accuracy %**	**Precision %**	**Recall %**	**F1 score %**
10	85.91	84.66	84.86	84.73
20	86.99	86.73	84.29	85.28
**50**	**88.39**	**86.62**	**85.87**	**86.22**
100	87.34	86.13	86.05	86.07

In parameter experiments, bold values indicate the best classification results are obtained for the parameters at their current values.

**Table 2:** Table of average performance results generated by adjusting the parameter Hop Length model in the Fourier transform.

**Hop-length**	**Accuracy %**	**Precision %**	**Recall %**	**F1 score %**
**1**	**88.39**	**86.62**	**85.87**	**86.22**
5	88.47	86.49	86.26	86.37
10	86.66	85.69	85.38	85.53
20	86.56	85.67	84.89	85.25

In parameter experiments, bold values indicate the best classification results are obtained for the parameters at their current values.

**Table 3:** Table of average performance results generated by adjusting the parametric Mel filter number model.

**N-filter**	**Accuracy %**	**Precision %**	**Recall %**	**F1 score %**
6	82.44	82.47	80.10	80.49
**12**	**88.39**	**86.62**	**85.87**	**86.22**
24	82.98	82.55	79.66	80.71

In parameter experiments, bold values indicate the best classification results are obtained for the parameters at their current values.

**Table 4:** Table of average performance results generated by the sample rate model for adjusting the parametric Mel filter.

**Sampling rate**	**Accuracy %**	**Precision %**	**Recall %**	**F1 score %**
100	85.45	84.50	83.87	84.17
200	85.91	84.66	84.86	84.73
300	85.91	84.82	84.62	84.72
**400**	**88.39**	**86.62**	**85.87**	**86.22**
800	85.80	84.82	84.55	84.68

In parameter experiments, bold values indicate the best classification results are obtained for the parameters at their current values.

**Table 5:** Average performance results generated by patch size model in tuned parameter CNN.

**Patch size**	**Accuracy %**	**Precision %**	**Recall %**	**F1 score %**
100	84.02	83.72	83.10	83.39
**200**	**88.39**	**86.62**	**85.87**	**86.22**
400	86.67	84.70	84.33	84.54

In parameter experiments, bold values indicate the best classification results are obtained for the parameters at their current values.

**Table 6:** Table of optimal experimental parameters for the LogMel-CRNN model proposed in this paper.

**Experiment parameter**	
FT_kenel size	50
FT_Hop-Length	1
Mel-N-filter	12
Mel Sampling rate	400
CNN Patch size	200

**Table 7:** Four assessment indicators for classifying EEG signals into alert and fatigue states.

**Accuracy %**	**Precision %**	**Recall %**	**F1 Score %**
**Two-class: Vigilance or fatigue**			
88.39	86.62	85.87	86.22

**Table 8:** Four assessment indicators for classifying EEG signals into three states of alertness, fatigue, and drowsiness.

**Accuracy %**	**Precision %**	**Recall %**	**F1 Score %**
**Three-class: Vigilance, fatigue, or drowsiness**			
81.30	81.67	81.97	81.80

### 4.5. Comparison with existing methods

This section mainly discusses the differences between the proposed and related fatigue detection methods. Here, we have selected four existing methods for comparison: (1) LSTM (Hefron et al., 2017); (2) ESTCNN (Gao et al., 2019); (3) EEG_Conv (Zeng et al., 2018); (4) EEG_Conv_R (Zeng et al., 2018).

LSTM: In the literature (Hefron et al., 2017), a long short-term memory network, a particular type of RNN model, is proposed. The network considers the temporal correlation between improving features' smoothness and solving the problem of gradient disappearance and gradient explosion during backpropagation in simple recurrent networks. LSTM provides algorithms with fine-grained control over what is put into and removed from memory in hidden layers called memory cells. It does this through a combination of three gates: an input gate, a forgotten gate, and an output gate. The forgot gate determines when inputs are remembered or ignored in the hidden state through a dedicated mechanism. The network is adequate for the task of “long-term memory.” Driving fatigue detection is a type of long-term memory task. We use this method to compare with the one proposed in this paper, but also because it introduces a lot of content, resulting in more parameters, which makes training much more difficult.

ESTCNN: In this research (Gao et al., 2019) proposed a Spatio-temporal convolutional neural network (ESTCNN) based on EEG signals. The network introduces a core block to extract temporal correlations from the EEG signal, which consists of three convolutional blocks and a pooling layer. The combination of the core block and the dense layer is then used to learn valid information related to fatigue. The model is a 14-layer network consisting of three core blocks, two dense layers, and a softmax layer, respectively. The results show that the Spatio-temporal structure of the framework offers advantages in terms of computational efficiency and reference time.

EEG_Conv: In the literature (Zeng et al., 2018) the authors proposed two models for predicting the mental state of drivers in EEG signals. EEG data were collected on a driving simulation platform constructed themselves and applied to models EEG-Conv and EEG-Conv-R, respectively. Where EEG-Conv has a total of eight layers, consisting of an input layer, three convolutional layers, a pooling layer, a local response normalization layer, a fully connected layer, and an output layer. In the paper, the prediction performance of the proposed classifier is investigated for both within-subject and between-subject EEG data. Inter-subject prediction refers to training and test data from the same subject, while inter-subject prediction refers to training and test data from different subjects. The results show that the proposed method has better generality for detecting mental states from various subjects.

EEG_Conv_R: In the literature (Zeng et al., 2018), in order to further improve the classification accuracy based on the model EEG-Conv, researchers have combined EEG-Conv with residual learning and proposed the EEG-Conv-R model. Residual learning explicitly re-represents layers as learning residual functions concerning the layer inputs rather than learning unreferenced functions. In other words, the residual layer knows the changes in the perturbations. From the experimental results, EEG-Conv-R converges faster than EEG-Conv and takes less time to extract features in the training phase.

**Figure 7:**
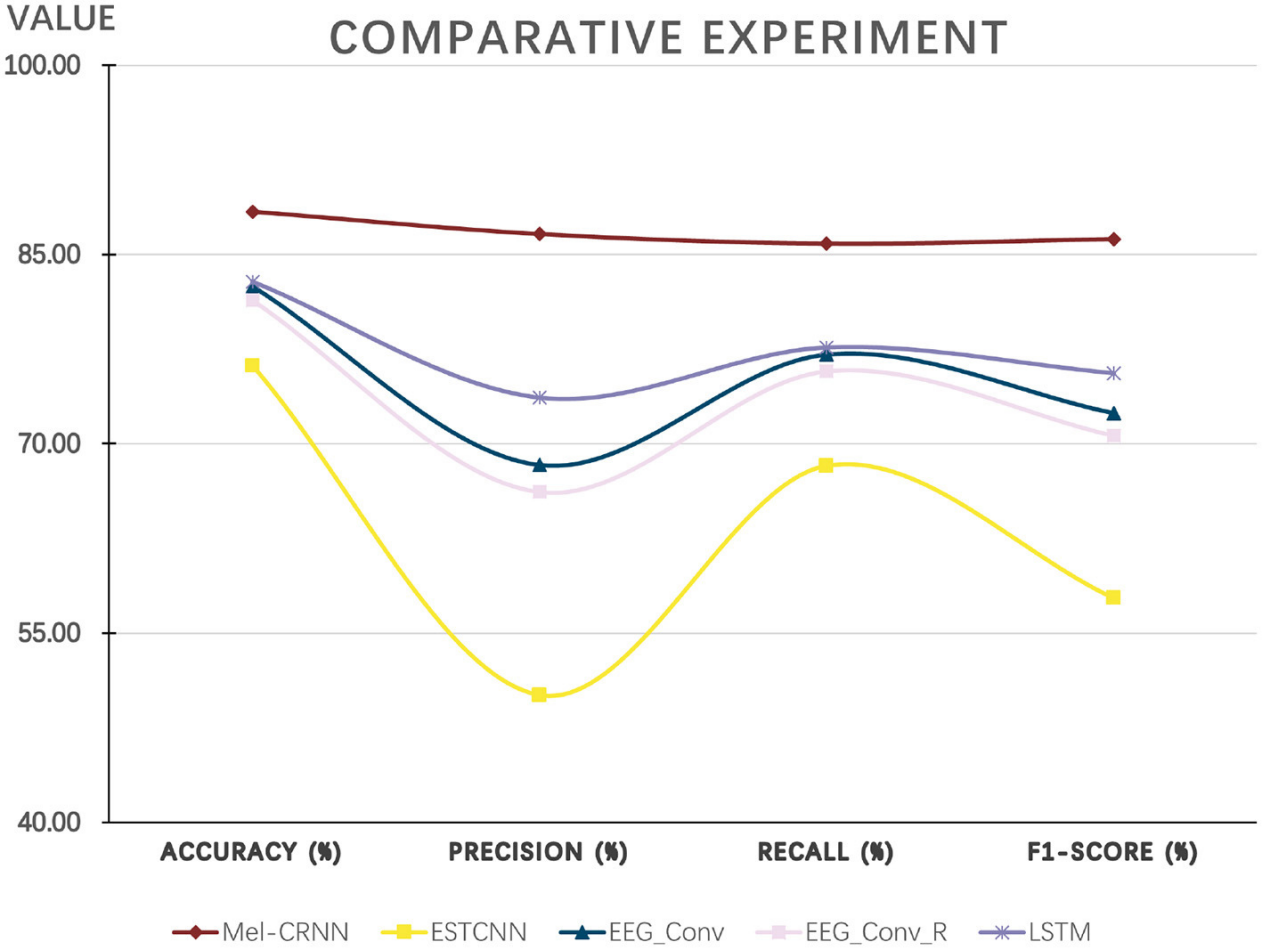
Experimental results compared with existing methods.

In Figure 7, the abscissa represents the four indicators of fatigue predicted by the model, and the ordinate represents the average value of each indicator. As seen in Figure 7, the method proposed in this paper outperforms the other four existing methods in all four indicators compared with the current four methods.

## 5. Conclusion

This paper proposes using Convolutional Recurrent Neural Network based on Log Mel Spectrogram (LogMel-CRNN) for driver EEG fatigue detection. In particular, we use one-dimensional convolution to compute STFT, which improves the accuracy of extracting features by 5.12% compared to the traditional STFT method. To build LogMel-CRNN, we investigated four model structures and obtained different recognition results for each of them, where the STFT of extracted EEG signals and log Mel spectrograms as features input to the model got the highest accuracy of 88.39%, the accuracy of 86.62%, recall of 85.87%, and F1 score of 86.22%, thereby the EEG-based fatigue detection model was thus finalized. The use of log-Mel spectrogram features was shown to improve classification accuracy and model performance in the tuning structure experiments. We demonstrate that the model outperforms several previous state-of-the-art methods through comparative experiments. In practical driving applications, lightweight EEG signal acquisition methods are an essential issue, as is noise handling for real-time fatigue detection. In the future, we will further optimize LogMel-CRNN to obtain better detection results for fatigue triple classification, to be able to implement the algorithm in practical applications as well as to extend it to more recognition tasks.

## Data availability statement

The original contributions presented in the study are included in the article/supplementary material, further inquiries can be directed to the corresponding author/s.

## Author contributions

DG: conceptualization, methodology, and writing. XT: methodology and writing. MW: methodology and data curation. GH: validation. YZ: supervision, conceptualization, and writing. All authors contributed to the article and approved the submitted version.
